# Identifying advanced MAFLD in a cohort of T2DM and clinical features

**DOI:** 10.3389/fendo.2023.1058995

**Published:** 2023-02-22

**Authors:** Ana Maria Sanchez-Bao, Alfonso Soto-Gonzalez, Manuel Delgado-Blanco, Vanesa Balboa-Barreiro, Diego Bellido

**Affiliations:** ^1^ Division of Endocrinology and Nutrition, Ferrol University Clinical Hospital, Ferrol, Spain; ^2^ Division of Endocrinology and Nutrition, A Coruña University Clinical Hospital, A Coruña, Spain; ^3^ Division of Gastroenterology and Hepatology, A Coruña University Clinical Hospital, A Coruña, Spain; ^4^ Epidemiology Department, A Coruña University Clinical Hospital, A Coruña, Spain

**Keywords:** MAFLD, NAFLD, NASH, T2DM, liver fibrosis, obesity, metabolic syndrome

## Abstract

**Background:**

MAFLD is the most common cause of chronic liver disease, affecting 25% of the global population. Patients with T2DM have an increased risk of developing MAFLD. In addition, patients with T2DM have a higher risk of advanced forms of steatohepatitis and fibrosis. Identifying those patients is critical in order to refer them to specialist and appropriate management of their disease.

**Aims and Objectives:**

To estimate advanced fibrosis prevalence in a cohort of patients with T2DM and to identify possible predictors.

**Methods:**

subjects with T2DM during regular health check-up were enrolled. Demographic and general characteristics were measured, including metabolic parameters and homeostasis model assessment of insulin resistance (HOMA2-IR). Four non-invasive fibrosis scores (NAFLD fibrosis scores, FIB-4, APRI, Hepamet fibrosis score) were measure and compared with transient elastography (TE).

**Results:**

96 patients (21%) presented risk of significant fibrosis (≥F2) measured by TE and 45 patients (10%) presented with risk of advanced fibrosis F3-F4. Liver fibrosis was related to BMI, AC, HOMA2-IR. The results of the non-invasive fibrosis scores have been validated with the results obtained in the TE. It is observed that the index with the greatest area under the curve (AUC) is APRI (AUC=0.729), with a sensitivity of 62.2% and a specificity of 76.1%. However, the test with better positive likelihood ratio (LR+) in our study is NAFLD fibrosis score.

**Conclusions:**

Our results show that in a general T2DM follow up, 10% of patients were at risk of advanced fibrosis. We found a positive correlation between liver fibrosis and BMI, AC and HOMA2-IR. Non-invasive fibrosis markers can be useful for screening, showing NAFLD Fibrosis score a better LHR+ compared to TE. Further studies are needed to validate these results and elucidate the best screening approach to identify those patients at risk of advanced MAFLD.

## 1 Introduction

Metabolic dysfunction-associated fatty liver disease (MAFLD), formerly named non-alcoholic fatty liver disease (NAFLD), is the most common cause of chronic liver disease, affecting 25% of the global population (1). It is nowadays a major health and economic burden worldwide. MAFLD is diagnosed in patients when they have both hepatic steatosis and any of the following three metabolic conditions: overweight/obesity, diabetes mellitus, or evidence of metabolic dysregulation (MD) in lean individuals (2).

While simple steatosis generally has a benign course, it is well known that advanced forms, especially when fibrosis is present, may progress to cause cirrhosis, liver failure and hepatocellular carcinoma (HCC) (3).

Patients with type 2 diabetes mellitus (T2DM) have an increased risk of developing MAFLD, with reported prevalence ranging from 49 to 74%. Moreover, patients with T2DM have a higher risk of developing advanced stages of steatohepatitis and fibrosis (4). On the other hand, patients without T2DM at the time of MAFLD diagnosis, run a high risk of future T2DM development (5).

MAFLD remains asymptomatic in a significant proportion of patients. Measurements of hepatic aminotransferase levels in plasma and liver ultrasonography are commonly used screening tools but lack sensitivity for diagnosis. Liver biopsy remains the gold standard however is not free of risk as it is an invasive procedure (6). On the other hand, non-invasive fibrosis scores and transient elastography (TE) can be a first-line tool to identify low-risk patients since they are easy to apply and highly available and reproducible, making it easier to identify those patients who do not need more advanced diagnostic methods (7). MAFLD patients with evidence of nonalcoholic steatohepatitis and advanced fibrosis are at markedly increased risk of adverse outcomes, including overall mortality, and liver-specific morbidity and mortality, respectively. Identification of this cohort of patients is paramount, given the associated poorer outcomes, to target resources to those who need it most (1).

The aim of the study is to estimate the prevalence of advanced fibrosis in a cohort of patients who attend an endocrinology clinic for their T2DM follow up and to identify possible predictors of advanced fibrosis.

## 2 Methods

### 2.1 Study design and population

We selected patients seen at the Department of Endocrinology of University Clinical Hospital of A Coruña (Spain) during 2016 who fulfilled the following inclusion criteria: a medically confirmed diagnosis of T2DM according the American Diabetes Association criteria and acceptance of participation in the study, signing the corresponding informed consent document.

Exclusion criteria were 1) patients with type 1 DM (T1DM), latent autoimmune diabetes in adults (LADA), monogenic diabetes and other types of diabetes rather than T2DM. 2) patients with alcohol consumption > 40 g/day in men and > 20 g/day in women. 3) coexistence of liver disease 4) treatment with hepatotoxic drugs.

The sample size was calculated to cover the primary objective, estimating advanced fibrosis, with acceptable precision by 95% confidence interval. Considering a prevalence of hepatic fibrosis between 5-25% in patients with T2DM, a sample size of 450 patients was calculated with a precision between 2- 4% respectively by 95% confidence interval.

The study was approved by the Ethics and Clinical Research Committee (register number 2016/172), in accordance with the Declaration of Helsinki. All clinical data were obtained from the Electronic Medical Record System of the University Clinical Hospital of A Coruña, Spain, and patient anonymity was preserved.

### 2.2 Demographic and clinical variables

Study parameters included demographic variables (age, sex), anthropometric variables (height, weight, BMI, abdominal circumference), past medical history, time form T2DM diagnosis, anti-diabetic medications, and non-anti-diabetic medications, glucose level, HbA1c, insulin level, lipidic profile (total cholesterol, LDL cholesterol, non-HDL cholesterol, HDL cholesterol and triglycerides), creatinine concentration, aspartate transaminase (AST) and alanine transaminase (ALT) levels. Insulin Resistance (IR) was determined by the Homeostasis Model Assessment of IR (HOMA2-IR). HOMA2-IR was calculated by the following formula: [plasma glucose (mg/dL) ∗ plasma insulin (μU/mL)]/405). The HOMA2-IR provides a surrogate estimate of IR (8) and a cut-off point of 3.8 was selected, based on previously studied populations with similar characteristics (9).

### 2.3 Risk of advanced fibrosis evaluation

#### 2.3.1 Risk ok fibrosis: Non-invasive fibrosis scores

AST/platelet ratio index (APRI) was calculated as follows: (AST level/AST upper level of normal/platelet counts) × 100, considering a result of <0.5 as low risk, a result between 0.5 and 1.5 as intermediate risk, and a result > 1.5 as high risk (10); FIB-4 as (age × AST level/platelet count × √ALT), considering a result < 1.3 as low-risk, a result between 1.3 and 2.67 as intermediate risk, and high risk a result > 2.67 (11); NAFLD fibrosis score as follows: [-1.675 + 0.037 × age (years) + 0.094 × BMI (kg/m^2^) + 1.13 × impaired fasting glucose/diabetes (yes = 1, no = 0) + 0.99 × AST/ALT ratio - 0.013 × platelet count - 0.66 × albumin], considering a result of < -1.455 as low risk, a result between -1.455 and 0.676 as intermediate risk, and a result > 0.676 as high risk (12). Hepamet score was calculated using a free online application (https://www.hepamet-fibrosis-score.eu/), considering a result < 0.12 as low risk, results from 0.12 to 0.47 as intermediate risk, and results above 0.47 as high risk (13).

#### 2.3.2 Fibrosis evaluation with transient elastography

All patients were studied with transient elastography (TE) (FibroScan; Echosens, Paris, France). TE was performed by a single operator (with experience in more than 1000 exams before the start of the study). M or XL probe was used, in the lobe right liver, through the intercostal spaces, in the supine position, with the right arm in maximum abduction, suspended breathing and after fasting for 2 hours, according to the Boursier et al. criteria (14). The examination was considered valid when the interquartile range (IQR) did not exceed 30% of the total value obtained and the reference values ​​that have been used are 7.0–8.1 kPa (F0-F1), 8.2–9.6 kPa (F2), 9.7–13.5 kPa (F3 or advanced fibrosis) and >13.6 (F4 or cirrhosis). These cutoff levels have been chosen as they are known to have a high positive predictive value to confirm the existence of clinically relevant fibrosis and cirrhosis, as has been shown in previous studies (15–17).

### 2.4 Statistical analysis

A descriptive study of the variables included in the work was carried out. For the quantitative variables, the estimate of the mean is provided, together with the standard deviation (SD). The qualitative variables are expressed as absolute value and percentage, with the estimation of their confidence interval at 95%.

The comparison of means between two groups was performed using the non-parametric Mann Whitney U test after checking for normality with the Kolmomorov Smirnov test. The association between qualitative variables will be concluded with the Chi-square statistic or Fisher’s test.

To determine possible factors related to the presence of fibrosis, a univariate logistic regression analysis was carried out.

In the case of the results of the FIB-4, NAFLD fibrosis score, APRI and Hepamet fibrosis score biochemical indices, the results were compared with the diagnosis of fibrosis using the M and XL probes, and the accuracy of these indices was calculated by the area under the ROC curves. In each case, the estimate of the cut-off points were assessed using the Youden index. The Youden index (IJ) is defined as the maximum vertical distance between the ROC curve and the diagonal or line change and is calculated as IJ= max (sensitivity + specificity -1). In turn, the parameters of sensitivity, specificity, predictive values, ​​and probability ratios (or likelihood ratios) were calculated to determine the validity of the procedures.

ROC curve comparison was performed following the procedure described in DeLong et al. (18), using the algorithm of Sun and Xu (19).

Statistical analysis was carried out with the IMB SPSS Statistics 24.0 and RStudio programs (2022.2.0.443 version).

## 3 Results

### 3.1 Study variables

Although 577 patients were included, 448 patients were finally analyzed. The loss of cases is due to a significant loss of data in those cases, and therefore they were not considered for the analysis. **Table 1** shows the demographic and clinical characteristics of the study population were 231 (51,56%) of participants were women. It is observed that the mean age at the time of the interview was 65 ± 7.8 years, with the minimum age observed being 37 years. Regarding the time of evolution of the disease, a mean time of approximately 13 years was observed, reaching 40 years in some cases, with an interquartile range between 7 and 17 years, and HbA1c levels were 7.0 ± 1.15% while mean BMI was 31.5 ± 5.36 kg/m2.

**Table 1:** Demographic and clinical characteristics.

	Mean (SD)
**Age (years)**	65.44 (7.81)
**Duration of T2DM (years)**	12.97 (8.57)
**Abdominal circumference (cm)**	105.60 (12.84)
**BMI (kg/m2)**	31.50 (5.36)
**DBP (mmHg)**	81.87 (10.22)
**SBP (mmHg)**	141.23 (17.89)
**HbA1c (%)**	7.00 (1.15)
**Total cholesterol (mg/dl)**	178.06 (36.03)
**HDL cholesterol (mg/dl)**	48.36 (12.55)
**LDL cholesterol (mg/dl)**	100.33 (30.21)
**Triglycerides (mg/dl)**	149.83 (79.71)
**AST (UI/L)**	22.57 (9.15)
**ALT (UI/L)**	26.96 (14.84)
**HOMA2IR**	6.17 (14.03)
**FIB4 score**	1.37 (0.75)
**NAFLD Fibrosis Score**	-0.17 (1.05)
**APRI score**	0.27 (0.18)
**Hepamet score**	0.20 (0.15)

Results showed as mean (standard deviation).

ALT, alanine aminotransferase; AST, aspartate aminotransferase; BMI, body max index; DBP, diastolic blood pressure; HDL cholesterol, high-density lipoprotein cholesterol; HOMA2IR, Homeostasis Model Assessment 2IR; LDL, low-density lipoprotein cholesterol; SBP, systolic blood pressure; T2DM, type 2 diabetes mellitus.

Findings of TE show that 96 patients (21%) presented risk of significant fibrosis (≥F2) measured by TE and 45 patients (10%) presented with risk of advanced fibrosis F3-F4 (see **Table 2**).

**Table 2:** Results of Transient Elastography.

Transient Elastography (Fibroscan)	n (%)	CI 95%	
F0	201 (44.90)	40.29	49.51
F1	151 (33.70)	29.32	38.08
F2	51 (11.40)	8.46	14.34
F3	28 (6.30)	4.05	8.55
F4	17 (3.80)	2.03	5.57
Total valid tests	448 (100)		

Results showed absolute frequencies (percentage).

### 3.2 Factors associated to fibrosis

**Table 3** shows the factors associated with liver fibrosis measured with TE. It can be highlighted that body mass index (BMI) was identified as a risk factor (p=0.013); patients with a higher BMI have a higher risk of fibrosis. In relation to normal weight patients there was no increased risk in the overweight category, but there was an increased risk of advanced fibrosis in patients with obesity in all categories, so that patients with type I obesity have an OR of 4.95 (95%CI 1.42, 17.29), increasing this estimate of OR up to grade III-IV obesity, with an OR 9 (95%CI 1.62, 50.12). Abdominal circumference (AC) shows a similar behavior, with higher levels being observed in patients with fibrosis (108.99 ± 11.02 *vs.* 101.93 ± 10.74 cm, p<0.001), obtaining an OR=1.059 CI95% (1.028-1.09).

**Table 3:** Factors associated to hepatic fibrosis by TE.

	F0-F1-F2	F3-F4		
Variable	n=403	n=45	OR (95% CI)	p-value
Anthropometric Data				
**Abdominal circumference (cm)**	101.9 (10.74)	109,0 (11,02)	1,06 (1,03; 1,09)	<0,001
**BMI (kg/m^2)**	29.9 (4.35)	33.0 (5.31)	1.13 (1.07; 1.21)	<0.001
Normal weight - Overweight class I	99 (24.90%)	3 (7.50%)	Ref	
Overweight class II	123 (31.50%)	9 (22.50%)	2.42 (0.64; 9.16)	
Obesity class I	120 (30.70%)	18 (45%)	4.95 (1.42; 17.29)	
Obesity class II	38 (9.70%)	7 (17.50%)	6.08 (1.49; 24.73)	
Obesity class III – IV	11 (2.80%)	3 (7.50%)	9 (1.62; 50.12)	
Biochemical parameters				
**Total Cholesterol (mg/dl)**	181.41 (37.10)	164.46 (25.6)	0.98 (0.97; 0.99)	0.004
**HDL Cholesterol (mg/dl)**	49.37 (12.76)	42.16 (10.74)	0.94 (0.91;0.97)	<0.001
**LDL Cholesterol (mg/dl)**	103.15 (30.57)	87.17 (22.15)	0.97 (0.96; 0.99)	0.002
**Triglycerides (mg/dl)**	148.23 (82.27)	180.54 (89.20)	1.02 (1,01; 1,03)	0.009
**HbA1c (%)**	6.96 (1.17)	7.15(0.91)	1.15 (0.86;1.52)	0.092
HbA1c ≤ 7.5%	251 (73.60%)	22 (59.50%)	1	
Hb1Ac>7.5%	90 (26.40%)	15 (40.50%)	1.902 (0.94;3.80)	
**HOMA2IR**	5.706 (14.05)	7.07 (6.76)	1.01 (0.98;1.02)	<0.001

Quantitative variables are expressed as mean and SD and qualitative values as percentages.

BMI, body max index; HDL cholesterol, high-density lipoprotein cholesterol; HOMA2IR, Homeostasis Model Assessment 2IR; LDL, low-density lipoprotein cholesterol.

Total cholesterol, on the other hand, is shown to be a protective factor, as total cholesterol and HDL increase, the risk of fibrosis decreases. The opposite occurs with LDL cholesterol; higher levels of LDL cholesterol are related with hepatic fibrosis severity. Concerning insulin resistance, measured by HOMA2-IR, we found statistically significant correlation with the risk of liver fibrosis with an increase of 1.36 for patients with a degree of fibrosis F3-F4. Further details are shown in **Table 3**. Conversely, we found no evidence of significant statistical association between hepatic fibrosis and age, sex, hypertension or HbA1c levels (data not shown).

Regarding the non-invasive fibrosis scores, FIB-4, NAFLD Fibrosis Score, APRI and Hepamet Fibrosis Score were measured (see **Table 4**). We found that FIB-4 and APRI showed a statistically significant relationship with the TE in this cohort of patients. Those patients with higher FIB4 index, have 1.43 times more risk (OR=1.43, CI95%= (1.0-22.19)) while patients with higher levels of APRI have 12 times more risk (OR=12.59, CI95%= (2.60; 60.89)) of advanced fibrosis.

**Table 4:** Results of noninvasive fibrosis scores compared to TE.

	F0-F1-F2	F3-F4		
	n=403	n=45	OR (95% CI)	p-value
Non-invasive fibrosis scores				
**FIB4 score**	1.33 (0.72)	1.67 (1.09)	1.43 (1.01; 2.01)	0.049
**NAFLD Fibrosis Score**	0.34 (0.99)	0.04 (1.31)	1.34 (0.94; 1.92)	0.419
**APRI**	0.24 (0.17)	0.38 (0.25)	12.59 (2.60; 60.89)	<0.001
**Hepamet**	0.19 (0.14)	0.22 (0.19)	3.34 (0.43-25.79)	0.51

**Figure 1:**
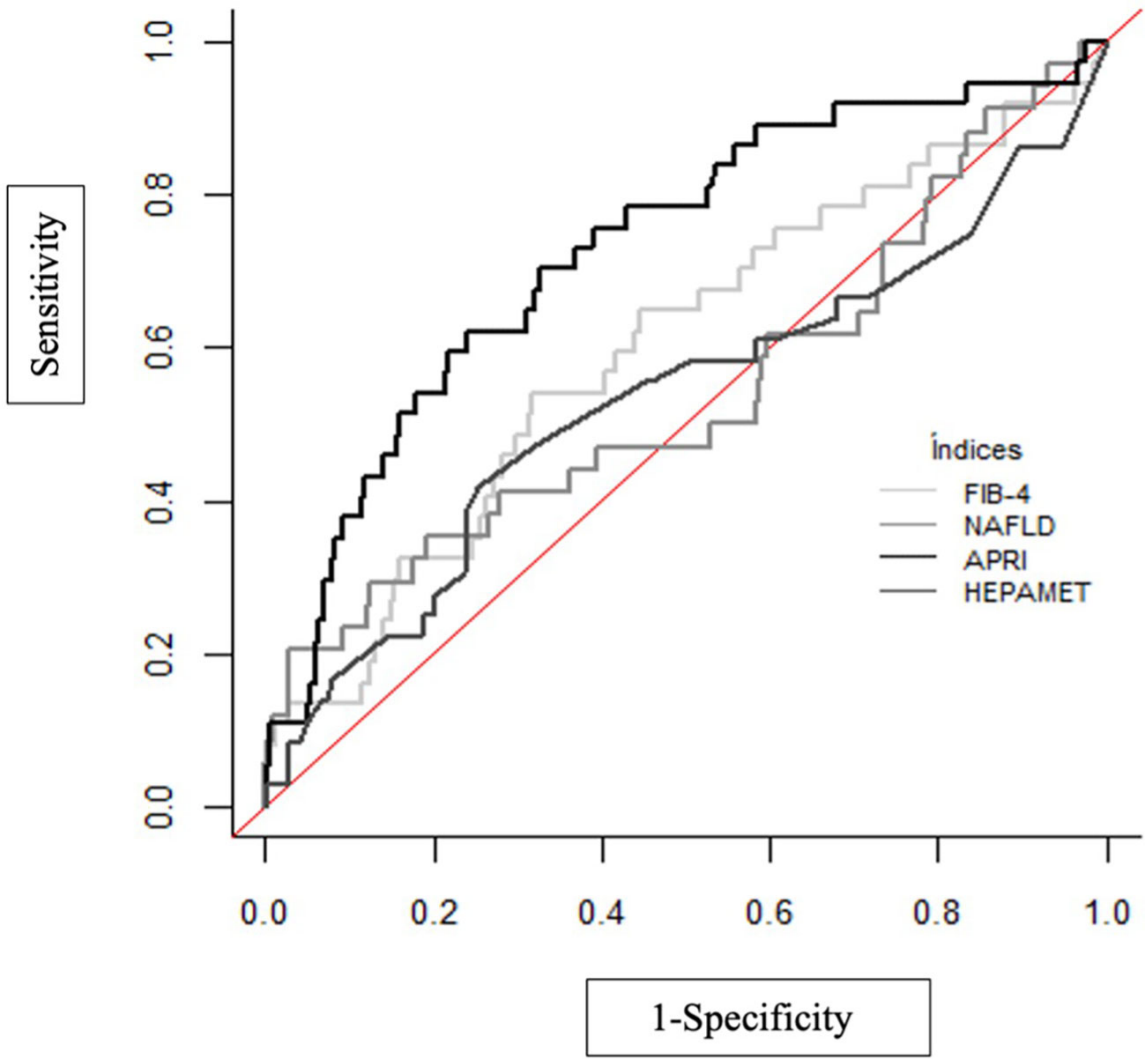
ROC curves for non-invasive fibrosis scores to TE.

The results of the non-invasive fibrosis scores have been validated with the results obtained in the TE (**Table 5**; **Figure 1**). It is observed that the index with the greatest area under the curve (AUC) is APRI (AUC=0.729), with a sensitivity of 62.2% and a specificity of 76.1% and the test with higher maximum positive likelihood ratio (LR+: 7.45) in this study was NAFLD fibrosis score. **Table 6** shows the pairwise comparisons of ROC curves (AUC) between these four tests for the diagnosis of fibrosis.

**Table 5:** Accuracy evaluation of non-invasive fibrosis scores compared to TE.

	FIB-4			NAFLD Fibrosis score			APRI			HEPAMET fibrosis score		
	mean	CI 95%		mean	CI 95%		mean	CI 95%		mean	CI 95%	
**AUC**	**0.59**	0.49	0.70	**0.54**	0.42	0.65	**0.72**	0.63	0.82	**0.53**	0.41	0.64
**Sensitivity**	**0.54**	0.37	0.70	**0.20**	0.08	0.35	**0.62**	0.45	0.78	**0.41**	0.25	0.58
**Specificity**	**0.68**	0.63	0.73	**0.97**	0.95	0.98	**0.76**	0.71	0.80	**0.74**	0.70	0.79
**NPV**	**0.93**	0.90	0.95	**0.92**	0.91	0.93	**0.94**	0.92	0.96	**0.92**	0.90	0.94
**PPV**	**0.15**	0.11	0.20	**0.44**	0.21	0.68	**0.22**	0.16	0.27	**0.15**	0.09	0.20
**LR+**	**1.71**			**7.45**			**2.60**			**1.64**		
**LR-**	**0.67**			**0.81**			**0.49**			**0.78**		

AUC, area under curve; Sensitivity, Specificity; NPV, negative predictive value; PPV, positive predictive value; LR+, positive likelihood ratio; LR-, negative likelihood ratio. Using the 4 non invasive test: FIB-4, NALFD fibrosis score, APRI, Hepamet Fibrosis score. Data are shown in mean and standard deviation.

**Table 6:** Contrast between different non-invasive fibrosis scores.

p-valor	NAFLD fibrosis score	APRI	HEPAMET fibrosis score
FIB-4	0.22	<0.001	0.08
NAFLD Fibrosis Score		<0.001	0.56
APRI			<0.001

P-values for pairwise comparisons.

## 4 Discussion

In this observational study, among a population of T2DM patients with an average glycemic control of HbA1c 7% and average duration of disease of 13 years, 21% of patients had risk of significant hepatic fibrosis (TE ≥ F2) in 10% of patients there was suspicion of advanced hepatic fibrosis (TE ≥ F3), being consistent with similar studies (20). Neither control of diabetes nor time of evolution were predictors of advanced fibrosis, consistent with what has been described previously (21).

We found that hepatic fibrosis was associated to BMI and to AC with a growing trend, being in tune with known studies (22). Obesity is a known major risk factor for both hepatic steatosis and fibrosis, since adipose tissue dysfunction causes intrahepatic triglyceride accumulation through increased hepatic lipid flow, IR and pro-inflammatory adipokines release; moreover the oxidative stress and inflammation associated with excess adiposity promotes dysregulation of the genes involved in liver tumorigenesis, increasing the risk of hepatocarcinoma (23). This seems to be related to the distribution of body fat, presenting those patients more visceral adiposity. In this study we found that as the AC increases by one unit, the risk of hepatic fibrosis increases by 6%. AC is predictive of increased visceral adipose tissue (VAT) among people with the same BMI and has been shown to be more strongly associated with amount of VAT than waist‐to‐hip ratio (24). There has been increasing interest in recent years in the role of VAT in MAFLD. Studies have demonstrated that VAT, which was originally considered a passive depot for energy storage, is an active endocrine tissue that releases many peptides and hormones that regulate metabolism, inflammation, and immunity, thus participating in the pathogenesis of MAFLD (25). In line with that, we found that HOMA2-IR is associated with the presence of advanced hepatic fibrosis in adults with T2DM, and it is known that HOMA2-IR has a significant positive correlation to visceral adipose tissue (26, 27).

Concerning the non-invasive fibrosis scores, they are a widely available, inexpensive, tool for first line identification of patients at risk. Nonetheless, data are still emerging regarding the optimal way to use these tests. Due to the generally low pretest probability of advanced fibrosis and cirrhosis in the general population, the positive predictive value (PPV) of a result above the high cut-off is typically modest, and often not sufficient to be diagnostic in the absence of additional supportive clinical information. In contrast, the negative predictive value (NPV) is generally very high, allowing the clinician to be confident that advanced fibrosis or cirrhosis has been excluded (28).

In this study, although APRI has a better AUROC related to TE, also the higher estimation of OR. On the other hand, NAFLD fibrosis score has the higher LR+, showing a theoretical better result for a screening test. Nevertheless, these tests are still being refined and recent diagnostic algorithms propose a two steps screening, using TE in second line to confirm those patients at risk of advanced fibrosis (29).

The large global impact of MAFLD and T2DM on healthcare systems requires a paradigm shift to early identification and risk stratification of MAFLD in primary care and diabetes clinics. Establishing a diagnosis may be especially important in patients with T2DM, not only because of its high prevalence, in this described cohort a 10% of patients with F3-F4 score of fibrosis, but also because it has been shown that patients with liver fibrosis and T2DM are at a high risk of serious hepatic pathology, including cirrhosis and hepatocellular carcinoma, increasing all-cause and liver-related mortality and morbidity, even after adjustment for potential confounding factors (30). In addition, a recent American Heart Association (AHA) Statement highlights that it may potentially worsen cardiovascular disease risk (CVD), independently of other components of the metabolic syndrome (31). Thus, the identification of MAFLD in this population may have important management implications beyond hepatic disease, including intensive therapy to reduce CVD risk.

This study has several limitations. Firstly, the diagnosis of hepatic fibrosis was based on TE rather than liver biopsy, which may have resulted in misclassification of some patients. Liver biopsy remains the gold standard for MAFLD diagnosis. However, cost, procedure related complications, and intra- and inter-observer variations in reporting the histology are the major draw backs of liver biopsy, and, therefore, it is usually not recommended in clinical practice for general screening (6). Secondly, although our study had sufficient power to identify a significant association of hepatic fibrosis with anthropometric data as BMI and AC, we lack body composition data. Further studies analyzing total body fat and abdominal fat would be interesting, as recent data suggest they are strongly related to the risk of MAFLD (32).

This study has also strengths. We used data from a well-characterized sample of T2DM patients, based on direct measurements collected by Endocrinologist and Hepatologist in a tertiary-level hospital in Spain. In addition, the most appropriate TE probe was selected in each patient (M or XL probe) to avoid measurement errors.

## 5 Conclusion

In conclusion, our results show that in a cohort of T2DM patients, 10% were at risk of advanced fibrosis. We found a positive correlation between liver fibrosis and BMI, AC and HOMA2-IR. Non-invasive fibrosis markers can be useful for screening, showing NAFLD Fibrosis score a better LR+ compared to TE. Further studies are needed to validate these results and elucidate the best screening approach to identify those patients at risk of advanced MAFLD.

## Data availability statement

The raw data supporting the conclusions of this article will be made available by the authors, without undue reservation.

## Ethics statement

The study was conducted according to the guidelines of the Declaration of Helsinki and approved by the Autonomic Committee of Clinical Research Ethics of Galicia, Spain (register number 2016/172). The authors also certify that formal approval to conduct the experiments described has been obtained from the human subjects review board of their institution and could be provided upon request. The participants did not receive economic profit. Informed consent was obtained from all subjects involved in the study. Written informed consent has been obtained from the patients to publish this paper.

## Author contributions

All authors listed have made a substantial, direct, and intellectual contribution to the work and approved it for publication.
